# Productivity trends and collaboration patterns: A diachronic study in the eating disorders field

**DOI:** 10.1371/journal.pone.0182760

**Published:** 2017-08-29

**Authors:** Juan-Carlos Valderrama-Zurián, Remedios Aguilar-Moya, Antonio Cepeda-Benito, David Melero-Fuentes, María-Ángeles Navarro-Moreno, Asunción Gandía-Balaguer, Rafael Aleixandre-Benavent

**Affiliations:** 1 Universidad Católica de Valencia "San Vicente Mártir", Instituto de Documentación y Tecnologías de Información, Valencia, Spain; 2 Universidad Católica de Valencia "San Vicente Mártir", Departamento de Ciencias de la Educación, Godella, Spain; 3 The University of Vermont, Department of Psychological Science, Burlington, Vermont, United States of America; 4 Universidad Católica de Valencia "San Vicente Mártir", Departamento de Ciencias Experimentales y Matemáticas, Valencia, Spain; 5 Universidad Católica de Valencia "San Vicente Mártir", Departamento Ciencias Aplicadas a la Actividad Física y Gestión, Valencia, Spain; 6 INGENIO (CSIC-Universidad Politécnica de Valencia) & UISYS (CSIC-Universidad de Valencia), Valencia, Spain; Katholieke Universiteit Leuven Rega Institute for Medical Research, BELGIUM

## Abstract

**Objective:**

The present study seeks to extend previous bibliometric studies on eating disorders (EDs) by including a time-dependent analysis of the growth and evolution of multi-author collaborations and their correlation with ED publication trends from 1980 to 2014 (35 years).

**Methods:**

Using standardized practices, we searched *Web of Science* (WoS) *Core Collection* (WoSCC) (indexes: *Science Citation Index-Expanded* [SCIE], & *Social Science Citation Index* [SSCI]) and *Scopus* (areas: *Health Sciences*, *Life Sciences*, & *Social Sciences and Humanities*) to identify a large sample of articles related to EDs. We then submitted our sample of articles to bibliometric and graph theory analyses to identify co-authorship and social network patterns.

**Results:**

We present a large number of detailed findings, including a clear pattern of scientific growth measured as number of publications per five-year period or quinquennium (Q), a tremendous increase in the number of authors attracted by the ED subject, and a very high and steady growth in collaborative work.

**Conclusions:**

We inferred that the noted publication growth was likely driven by the noted increase in the number of new authors per Q. Social network analyses suggested that collaborations within ED follow patters of interaction that are similar to well established and recognized disciplines, as indicated by the presence of a “giant cluster”, high cluster density, and the replication of the “small world” phenomenon—the principle that we are all linked by short chains of acquaintances.

## Introduction

Eating disorders (EDs) are mental disorders with severe consequences [1–3]. The better known and possibly the most widely studied of the EDs are *anorexia and bulimia nervosa*, both of which were included in the DSM-III in 1980 [4,5]. *Binge eating disorder* (BED) was first covered in the DSM-IV in 1994 under the larger rubric of *eating disorder not otherwise specified* (EDNOS) [6–7]. BED has since become the most prevalent of the EDs [8] and is now considered a singular ED in the DSM-5 [1]. Other less common eating and feeding related EDs include *avoidant/restrictive food intake disorder*, *pica*, *rumination disorder*, and *unspecified feeding or eating disorder* [1].

Bibliometric studies can provide a window into the interest and research activity associated with specific scientific questions and medical phenomena. For example, Theander [9] used Medline and PsycINFO to study publication patterns related to EDs from 1960 to 1999 and found an almost 27-fold increase in the number of ED references over the 40-year period (which substantively outpaced the average 4-fold growth observed over the same period of time across all medical fields). Clinton [10] searched the database PsycINFO and replicated Theander’s [9] findings, but over a much larger period of time: 1900 to 2009. Clinton [10] also reported that, relative to other psychiatric disorders, the acceleration of research on EDs peaked in the 1980s and stagnated thereafter. Nonetheless, a recent bibliometric study on EDs, Soh and Walter [11] found substantive growth trends in Medline and PsycINFO from 1970 to 2011 (although these authors focused just on publications related to the cross-cultural aspects of EDs).

Given that research teams now dominate the impact of scientific work across various disciplines [12], we believe it would be important to examine the possible role or influence of scientific collaborations in the observed rapid growth of ED research activity. Given that connections between a set of coauthors can be represented as a social network, social network analysis (SNA) can be applied to study how scientific collaborations originate and evolve in parallel with research publications for one or several academic communities [13]. Therefore, the present study seeks to extend previous bibliometric studies on EDs by including a time-dependent analysis of the growth and evolution of multi-author collaborations and their correlation with ED publication trends. To this end, we propose to examine traditional bibliometric indices along with the use of graph theory, which allows for the examination of co-authorship patterns and social network analyses [14–18]. We will focus in the 35-year period starting in 1980, which corresponds to the noted relative peak of research activity acceleration on EDs [10]. In addition, we casted a wider search net than previous ED bibliometric studies by using a larger and more comprehensive set of citation and reference databases.

## Methods

### Search strategy and procedures

We selected *Web of Science* (WoS) *Core Collection* (WoSCC) (indexes: *Science Citation Index-Expanded* [SCIE], & *Social Science Citation Index* [SSCI]) and *Scopus* (areas: *Health Sciences*, *Life Sciences*, & *Social Sciences and Humanities*) to conduct our search. Our strategy aimed to retrieve the complete collection of articles related to EDs published between 1980–2014 in the health and social sciences by searching journals included in any of the categories or areas listed in Table 1 (WoS Category) or Table 2 (Scopus Subject Areas). To this purpose, we entered a broad collection of ED-related keywords in the fields “Title” or “Topic” (for *WoSCC*), as well as in the fields “Title” or “Authkey” or “Indexterms” (for *Scopus*). The keywords used were: “eating disorder*”, or “pica”, or "rumination disorder*", or "avoidant food intake disorder*", or "restrictive food intake disorder*", or "anorexia nervosa*", or "bulimia nervosa*", or "binge eating disorder*". Each of the data based utilized allow for narrowing the search to specific document types. In both cases we limited our search to “Articles”. The search also excluded any articles that contained any of the 223 animal and insect terms included in the *Agricultural Thesaurus and Glossary* of the *National Agricultural Library* of the United States Department of Agriculture (agclass.nal.usda.gov). The exclusionary terms were entered in the *Topic* field of the *WoSCC* and in the *Title-Abs-Key* field of *Scopus*. This search strategy yielded 18,996 bibliographic records from *WoSCC* and 22,516 from *Scopus*.

**Table 1:** Health Sciences and Social Sciences from WoS Categories (SCIE & SSCI).

**SCIE**			
Allergy; Anatomy & Morphology; Andrology; Anesthesiology; Behavioral Sciences; Biochemical Research Methods; Cardiac & Cardiovascular Systems; Chemistry, Medicinal; Clinical Neurology; Critical Care Medicine; Dentistry, Oral Surgery & Medicine; Dermatology; Education, Scientific Disciplines; Emergency Medicine;	Endocrinology & Metabolism; Engineering, Biomedical; Gastroenterology & Hepatology; Genetics & Heredity; Geriatrics & Gerontology; Health Care Sciences & Services; Hematology; Immunology; Infectious Diseases; Integrative & Complementary Medicine; Medical Ethics; Medical Informatics; Medical Laboratory Technology;	Medicine, General & Internal; Medicine, Legal; Medicine, Research & Experimental; Microbiology; Nanoscience & Nanotechnology; Neuroimaging; Neurosciences; Nutrition & Dietetics; Obstetrics & Gynecology; Oncology; Ophthalmology; Orthopedics; Otorhinolaryngology; Pathology; Pediatrics; Peripheral Vascular Disease;	Pharmacology & Pharmacy; Physiology; Primary Health Care; Psychology; Radiology, Nuclear Medicine & Medical Imaging; Reproductive Biology; Respiratory System; Rheumatology; Sport Sciences; Surgery; Toxicology; Transplantation; Tropical Medicine; Urology & Nephrology; Virology
**SSCI**			
Anthropology; Area Studies; Communication; Criminology & Penology; Cultural Studies; Demography; Economics; Education & Educational Research;	Education, Special; Ethics; Ethnic Studies; Family Studies; Gerontology; Health Policy & Services; Hospitality, Leisure, Sport & Tourism; Law;	Political Science; Psychology, Applied; Biological; Clinical; Developmental; Educational; Experimental; Mathematical; Multidisciplinary; Psychoanalysis;	Social; Social Issues; Social Sciences, Biomedical; Social Work; Sociology; Urban Studies; Women's Studies.
**WoS Categories used both in SCIE as in SSCI**			
Nursing; Psychiatry;	Public, Environmental & Occupational Health		Rehabilitation; Substance Abuse.

**Table 2:** Health Sciences and Social Sciences from Scopus Areas.

Health Sciences	Life Sciences	Social Sciences & Humanities
Dentistry Health Professions Medicine Nursing	Biochemistry, Genetics & Molecular Biology Neuroscience Immunology & Microbiology Pharmacology, Toxicology & Pharmaceutics	Psychology Social Sciences

The search yielded a total of 41,512 bibliographic records that were included in a relational database to purge redundancies and false positives. Following the pre-stablished procedures described elsewhere (see [19]), a total of 16,789 records were removed due to duplications and overlapping (n = 9,343) or false positives (n = 7,446). The signature variations from single authors of the final sample of 24,723 records were then standardized through a manual review of all signatures, checking institutional affiliations in case of doubt. Our sample is the broadest, most comprehensive, and inclusive to date for the period covered. That is, our approach retrieved almost twice as many references as the second largest bibliometric analysis on ED to date (see Table 3).

**Table 3:** Characteristics of ED bibliometric studies conducted to date.

Paper	Searched Databases	Date range (*n* years)	*N* Publications
This paper	WoSCC (SCI-E & SSCI) + Scopus (includes Medline)	1980–2014 (35)	24,723
[11]	Medline + PsycINFO	1970–2011 (32)	1,417
[20]	Medline + PsycINFO	2006–2010 (5)	918
[21]	6 ED journals + 6 Psychiatry Journals	1996–2010 (15)	4,663
[22]	Medline + PsycINFO	1965–1999 (35)	13,768
[9]	Medline + PsycINFO	1960–1999 (40)	13,965
[10]	PsycINFO	1900–2009 (109)	< 10,000

### Bibliometric and social network analysis

To facilitate the time-dependent evolution of the volume of research activity and other bibliometric characteristics, we collected data on 30 different bibliometric and network measures for each of the seven, 5-year periods, or Quinquennials (Q) of the 1980–2014, time span (see Table 4).

**Table 4:** Bibliometric and network measures.

**Bibliometric measures**	
*n articles*	number of articles published
*% Q change*	% increase or decrease in number of articles from previous 5-year period
*n authors*	number of authors with at least one published article
*% authors > 9 articles*	% of authors with more than 9 published articles (a.k.a. as "highly productive authors"; see [23]).
*% authors 2–9 articles*	% of authors with 2 to 9 articles
*% authors = 1 article*	% of authors with a single article (a.k.a., "transitory authors" or “transience index”, see [24])
*n newcomers*	Number of authors with no publications during the previous Q (Q-start)
*n terminators*	Number of authors with at least one publication in the previous Q (Q-start) but no publications in the current Q (Q-end).
*n continuants*	Number of authors with publications in the previous and current Qs (Q-Start and Q-end, respectively)
*% collaborative articles*	% of articles with two or more authors
*% single authored articles*	% of articles that are single authored
*n signatures*	Sum of all signatures
*M (number of signatures per article)*	Collaboration index, or mean average number of signatures per article [25]
*SD (number of signatures per article)*	Standard deviation of the collaboration index
*maximum (range of number of signatures per article)*	Maximun number of signatures associated with a single article
*% authors (all articles in collaboration)*	% of authors whose articles were always co-authored
*% authors (all articles no in collaboration)*	% of authors whose articles were always single authored
*% authors (collaboration & no collaboration articles)*	% of authors whose articles were either co-authored or single authored
**Network measures**	
*n vertices*	Number of collaborating authors (authors with at least one co-author in at least one article)
*n isolate vertices*	Number of authors whose articles were always single authored.
*n edges*	Number of author pairs connected by one or more co-authorships.
*M edges/vertice*	Mean of the number of edges per vertex (excludes isolate vertices)
*SD edges/vertice*	Standard deviation of the mean of edges/vertex.
*network density*	The ratio of the number of linked vertices to the number of all possible links within any given network. All possible links between vertices represent a complete graph (K_n_) = n*(n– 1)/2 (where *n* is the number of vertices).
*Component*	Components are groups of vertices that are disconnected from each other. In a component, at least one vertex of each vertex pair is linked to another vertex within the component. Separate components do not have any vertices in common.
*average distance between vertices*	Mean length (number of edges) of all shortest paths from each vertex to all other vertices within the network (i.e., how many hop son average it takes to reach every other vertex)
*% edges = 1*	% of all the edges that link two vertices with a single article co-authorship between the pair
*% edges = 2–9*	% of all the edges that link two vertices with 2 to 9 articles co-authored between the pair
*% edges > 9*	% of all the edges that link two vertices with 10 or more articles co-authored between the pair
*n components*	Number of components.
*n vertices of the largest component*	Number of vertices in the largest component
*M (vertices per component)*	Average of vertices per component
*SD (vertices per component)*	Standard deviation of the average number of vertices per component.
*median (vertices per component)*	Median number of vertices per component.
*mode (vertices per component)*	Mode or most frequent number of vertices per component
*clustering coefficient*	The average of the local clustering coefficients of all the vertices [26]. The local clustering coefficient of a vertex quantifies the extent to which the vertices connected to that vertex are also connected to each other.
*Network Graphs*	Co-authorship networks were determined using the Force Atlas 2 algorithm [27], which enables analysis and visualization of networks *Gephi* (https://gephi.org/)

## Results

### Bibliometric results

Table 5 lists the diachronic progression of productivity measures per 5-year period, or Q. Overall, productivity, measured as the number of articles per Q increased by a factor of about 6 from Q1 (1980–1984) to Q7 (2010–2014). The final sample of 24,723 articles were authored or co-authored by 48,167 investigators, most of whom authored or co-authored a single manuscript (Transience Index = 73.89%, *n* = 35,593), with a substantive number of them authoring or co-authoring 2 to 9 articles (23.91%, *n* = 11,515), and very few authoring or co-authoring 10 or more publications (1.25%, *n* = 1,059). The substantive increase in the number of publications over the 35-year span does not appear to be due to a change in productivity measured categorically as the percentage of authors/co-authors with 1, 2 to 9, and more than 9 articles. That is, across Q2 to Q7, 76.85% to 78.47% of authors published a single article, 20.12% to 21.79% published between 2–9 articles, and 1.09% to 1.65% published 10 or more articles. Thus, the almost 6-fold growth in the number of publication in eating disorders over the 35-year span appears to be driven, on the surface, by the overall growth in the number of active authors per Q, which experienced an almost 10-fold increase from 2,111 in Q1 to 20,145 in Q7. Said differently, the growth of the number of authors/co-authors outpaced the growth of the number of total publications.

**Table 5:** Productivity measures.

*Measure*	*Q1*	*Q2*	*Q3*	*Q4*	*Q5*	*Q6*	*Q7*
	*1980–84*	*1985–89*	*1990–94*	*1995–99*	*2000–04*	*2005–09*	*2010–14*
*n* articles	1,207	1,918	2,180	2,758	3,914	5,432	7,314
% change	-	58,91	13,66	26,51	41,91	38,78	34,65
Transience Index (% authors with 1 article)	83.23	78.43	77.94	77.10	76.85	78.47	78.20
% authors with 2–9 articles	15.96	20.45	20.97	21.58	21.79	20.12	20.15
% authors with >9 articles	0.81	1.12	1.09	1.32	1.36	1.41	1.65
*n* authors	2,111	3,574	4,420	6,154	9,371	13,959	20,145
n author change	-	+1,463	+846	+1,734	+3,217	+4,588	+6,186
*% author change*	-	41%	19%	28%	34%	33%	31%
*n* newcomers	-	3,172	3,715	5,203	7,801	11,597	16,732
*% newcomers*	-	89%	84%	85%	83%	83%	83%
*n* continuants	-	402	705	951	1,570	2,362	3,413
*% continuants* (Q-start)	-	19%	20%	22%	26%	25%	24%
*% continuants* (Q-end)	-	11%	16%	15%	17%	17%	17%
*n* terminators	-	1,709	2,869	3,469	4,584	7,009	10,546
*% terminators*	-	81%	80%	78%	74%	75%	76%
articles:author	0,57	0,54	0,49	0,45	0,42	0,39	0,36

The number of authors per Q is the direct sum of newcomers (authors without a publication in the previous Q) plus continuants (authors with at least a publication in the previous and current Q). The number and percentage of newcomers and continuants are given in Table 5, which also shows the overall net gain from Q to Q. Table 5 shows a steady increase in the overall number of authors per Q of about 30% from Q to Q. This steady increase is the result of newcomers consistently outpacing terminators, and to a lesser extent a small but sustained increase in the percentage of continuants that remains from Q to Q (from 11% at Q2 to 17% at Q5, Q6 and Q7) that counteracts the small but sustained decline in the percentage of newcomers that appear from Q to Q (from a high of 89% at Q2 to a low of 83% at Q5, Q6 and Q7).

Table 6 lists the results per Q for each collaboration indicator. A total of 85.2% or 21,065 articles were co-authored by two or more authors, and only 14.8% or 3,658 articles were single authored. Four authors per article was the average over the 35-year period (*M* = 4.03, *SD* = 3.11). The percentage of co-authored articles increased with each new Q, from 61.97% in Q1 to 91.73% in Q7. Likewise, the average size and size range of the collaborating teams, or number of authors per article, also increased with each Q, from a low of *M* = 2.43 (*SD* = 1.67; range = 1 to 10) at Q1 to a peak of *M* = 4.79 (*SD* = 3.81; range = 1 to 176) in Q7.

**Table 6:** Collaboration measures.

*Measure*	*Q1*	*Q2*	*Q3*	*Q4*	*Q5*	*Q6*	*Q7*
	*1980–84*	*1985–89*	*1990–94*	*1995–99*	*2000–04*	*2005–09*	*2010–14*
% collaborative articles	61.97	72.21	77.57	83.18	87.63	88.51	91.73
% single authored articles	38.03	27.79	22.43	16.82	12.37	11.49	8.27
*n* signatures	2,927	5,559	7,011	9,981	15,779	23,358	34,998
*M* (signatures per article)	2.43	2.90	3.22	3.62	4.03	4.30	4.79
*SD* (signatures per article)	1.67	1.88	2.05	2.34	2.91	3.14	3.81
maximum (range signatures per article)	10	13	17	23	80	98	176
% authors with only co-authors articles	79.20	83.07	86.04	89.24	92.00	93.99	95.76
% authors with only single-authored articles	13.88	9.46	6.97	4.27	3.14	2.61	1.88
% authors with co- and single-authored articles	6.92	7.47	6.99	6.45	4.87	3.40	2.36

Similarly, the relative number of authors who engaged in collaborative efforts also increased from Q to Q, from a minimum 79.20% to a peak of 94,41% of authors publishing exclusively as co-authors in Q1 and Q7, respectively. Consequently, the percentage of authors publishing both single and co-authored articles, or only single authored publications, decreased considerably over time (see Table 6). In fact, only about 4% of authors between 2010 to 2014 could claim they had single authored a publication. Whereas the observed increases in collaboration rates and size of collaborating teams co-occurred with an increase in the total number of published articles noted earlier, the overall number or articles per author (articles:author) steadily declined from Q1 (0.57) to Q7 (0.36).

### Social network results

Table 7 shows the diachronic progression of co-authorships through network collaboration measures. The vast majority of authors (*n* = 46,472, 96.48%) was connected to the network at least within one of the seven 5-year periods. The number of vertices, or count of authors who are connected to at least to one other author in the network, increased from 1,818 in Q1 to 19,766 in Q7, with a mean average increase of 2,991 (*SD* = 2,045) vertices between consecutive Qs. Likewise, the total number of edges or connections that link any two vertices within the network, increased from 3,280 in Q1 to 93,618 edges in Q7, which corresponds to a mean average increase between consecutive Qs of 15,056 (*SD* = 15,295).

**Table 7:** Network measures.

*Measure*	*Q1*	*Q2*	*Q3*	*Q4*	*Q5*	*Q6*	*Q7*
	*1980–84*	*1985–89*	*1990–94*	*1995–99*	*2000–04*	*2005–09*	*2010–14*
*n* vertices	1,818	3,236	4,112	5,889	9,077	13,594	19,766
*n* isolate vertices	293	338	308	265	294	365	379
*n* edges	3,280	6,807	9,904	15,846	30,598	50,508	93,618
*M edges/vertex*	3.61	4.21	4.82	5.38	6.74	1.69	1.75
*SD* edges/vertex	2.77	3.68	4.35	5.22	9.63	2.75	3.47
network density	0.2679	0.1514	0.0146	0.0100	0.0045	0.0029	0.0019
average distance between vertices	2.794	3.580	6.875	7.781	6.148	6.167	5.383
% edges value = 1	90.67	84.75	85.88	83.21	83.77	87.30	87.64
% edges value 2–9	9.24	14.98	13.93	16.58	15.86	12.26	11.98
% edges value > 9	0.09	0.26	0.18	0.21	0.38	0.44	0.38
*n* components	414	588	623	817	1,085	1,509	1,901
*n* vertices of the largest component	110	180	1,151	1,769	3,684	5,894	9,992
*M* = (vertices per component)	4.39	5.50	6.60	7.21	8.37	9.01	10.40
*SD* = (vertices per component)	6.66	11.33	46.36	70.92	111.81	151.67	229.10
median (vertices per component)	3	3	3	4	4	4	4
mode (vertices per component)	2	2	2	3	2	2	2
clustering coefficient	0.936	0.917	0.913	0.914	0.915	0.917	0.918

Interestingly, the number of connections (edges) per author decreased over time, from *M = 3*.*61* (*SD = 2*.*77*) in Q1 to *M = 1*.*75* (*SD = 3*.*47*) in Q7 (although this average appears to have been stabilized over the last decade; *M* = 1.69 to 1.75; SD = 2.75 to 3.47). Consequently, network density decreased from 0.2679 at Q1 to 0.0019 at Q7.

The average, shortest distance between the vertices of the various network components initially increased from *M* = 2.79 to *M* = 7.78 edges (Q1 to Q4), but then decreased to *M* = 5.38 edges (Q4 to Q7). That is, it appears that from 1980 to 1999 collaboration networks grew with little interconnectivity among vertices, but from 2000 to 2014 the interconnectivity between co-authors increased.

Regarding vertices, the percentage of vertices per Q that shared a single article in common varied from about 83% to 91% but without following a time-line dependent pattern. Likewise, the proportion of vertices that authored between 2 to 9 articles jumped considerably across Qs without following an ascending or descending time pattern, from a minimum of 9.24% at Q1 and a maximum of 16.58% at Q4. The only time-dependent progression observed for productivity between vertex pairs was for those coauthors sharing 10 or more publications, which ranged between 0.09% and 0.26% during the first four Qs, and between 0.38% to 0.44% during the Qs representing the most recent 15 years.

The number of components and the number of vertices within the most populated component increased with each Q, from n = 414 components and n = 110 vertices (Q1) to n = 1,901 components and n = 9,992 vertices (Q7). Likewise, the average number of vertices per component increased from *M* = 4.39 (*SD* = 6.6) to *M* = 10.4 (*SD* = 10.4) from Q1 to Q7, respectively. In contrast, the other two measures of central tendency, the median and the mode, remained stable across Qs (Median ranged from 3 to 4; Mode ranged from 2 to 3). That is, whereas the mean average and the range of the number of vertices per component has increased considerably over time, the most common component or group size of connected authors has remained relatively small at about 3 vertices. The low number of vertices per component has resulted in high interconnectivity within the components, which have averaged clustering coefficients between 0.94 (Q1) and 0.91 (Q4).

**Fig 1:**
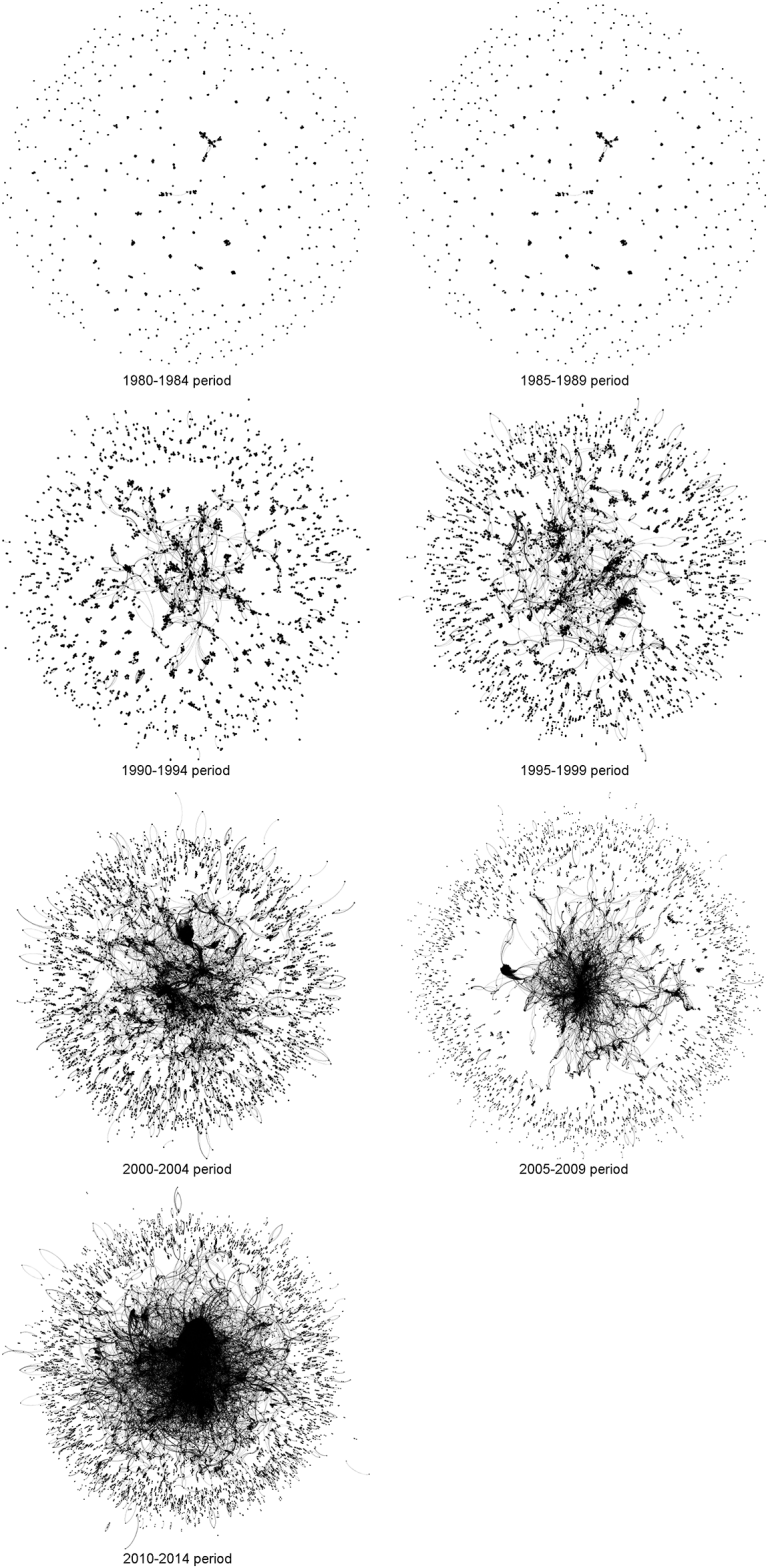
Network components evolution by Quinquennial.

Fig 1 depicts the components’ evolution of the social network Q by Q. Each vertex is represented by a gray dot and each pair of vertices are connected by a gray line. As the number of vertices in a component increases, and as the members within a component become more interconnected (denser) the dots become darker and larger in diameter. When vertices from different components connect with each other, they merge and become very large, and more central to the larger social network. The density (darkness) of the merged components increases as the density or interconnectivity between the vertices of the merged component increases. The figure clearly depicts the progressive increase in vertex density of the central components, as well as a relative increase in the density of components with few vertices per component in the periphery. In addition, Fig 1 shows that, starting with Q4, the central components began to connect with (absorb) the peripheral components.

**Fig 2:**
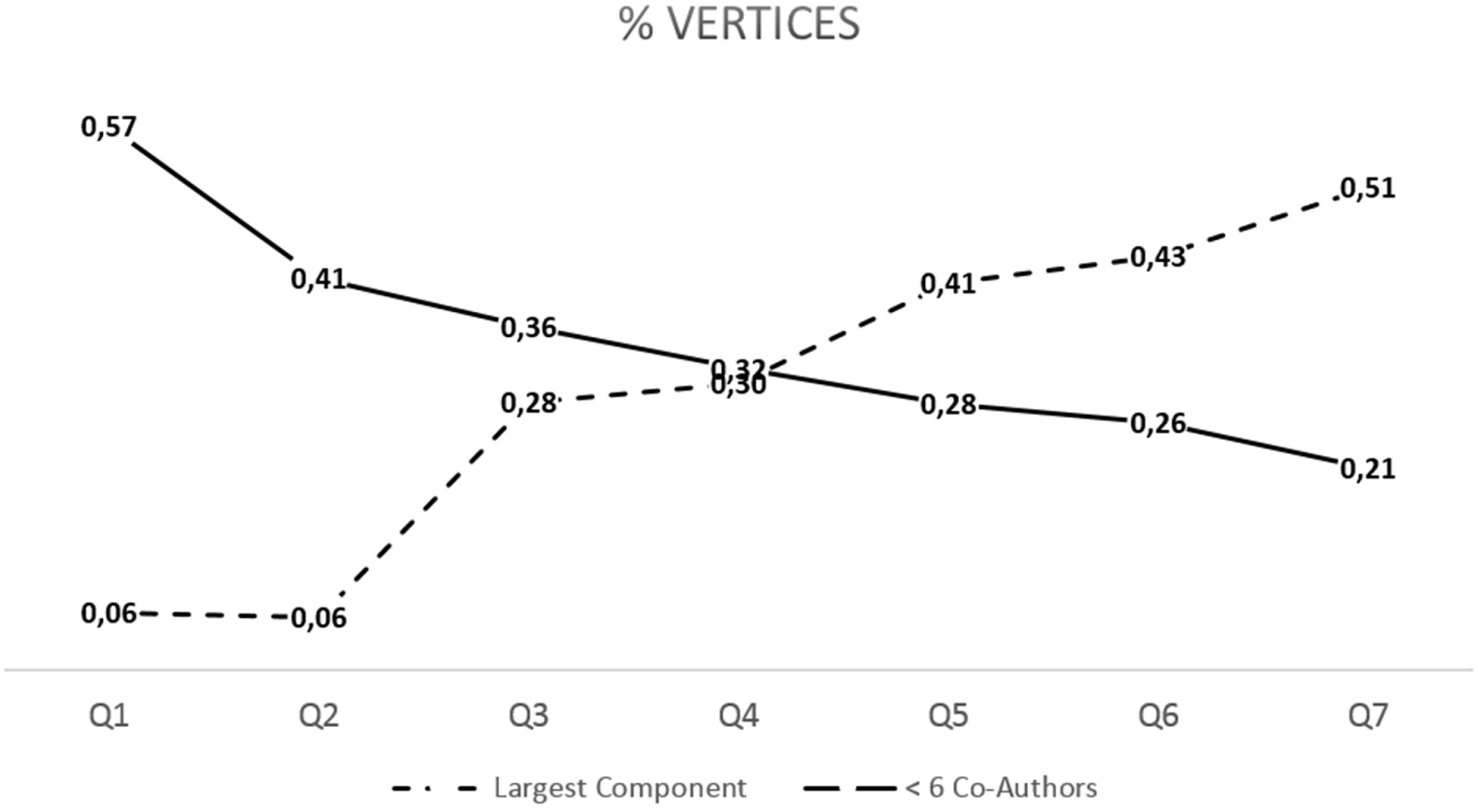
Within each Quinquennial, share of the total number of vertices taken by the largest component (dashed line) and the sum of components with 5 or fewer co-authors.

**Fig 3:**
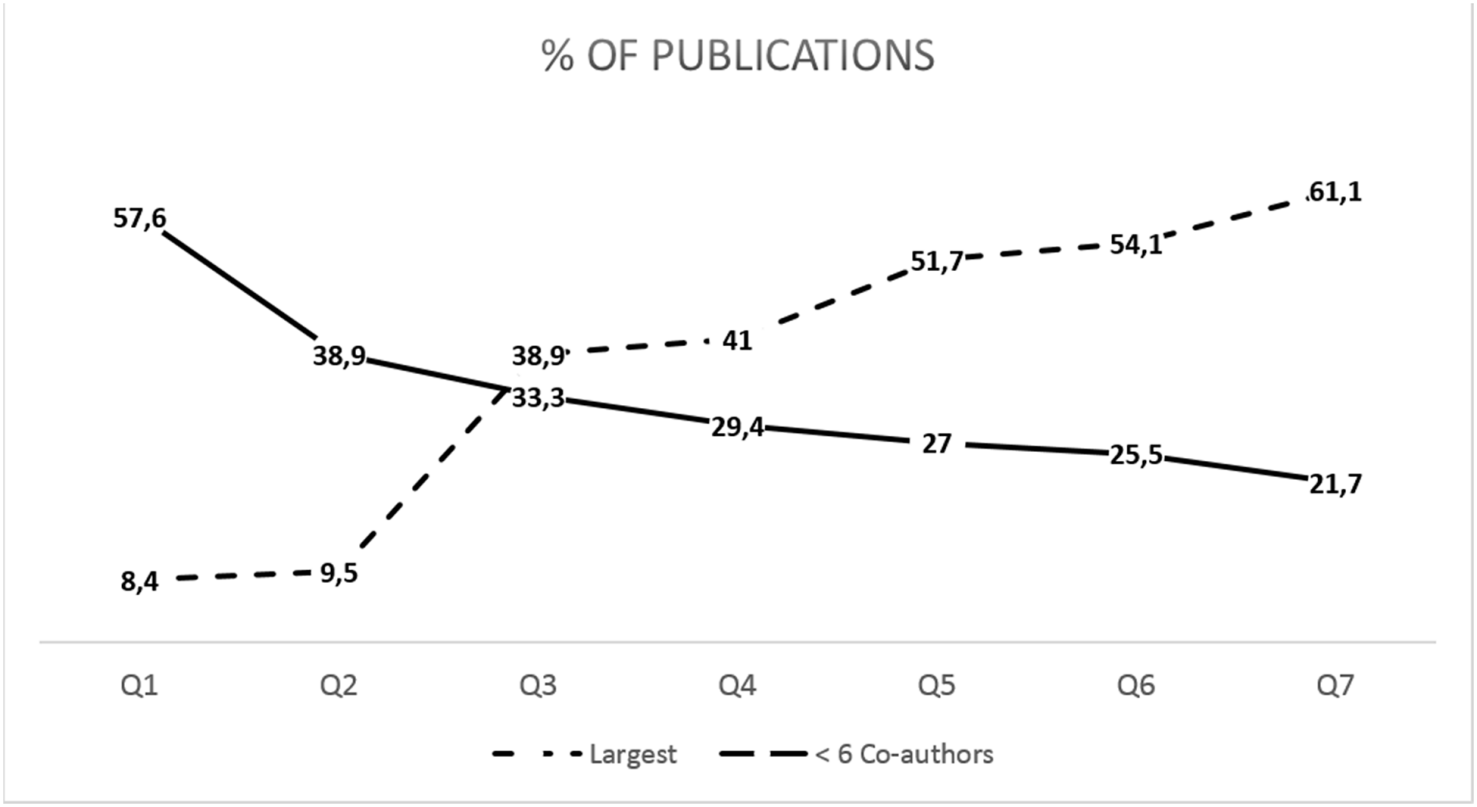
Within each Quinquennial, share of the total number of publications by the largest component (dashed line) and all components with 5 or fewer coauthors (solid line).

The distribution of components per vertex per Q revealed that with each successive Q, components become larger and larger components become more frequent. With time, the largest component eventually takes a much larger share of the universe of vertices within the network. For example, the largest component of the network takes up about 6% of all the vertices in Q1 and Q2; however, this proportion consistently increases Q to Q from 28% to 51% from Q3 to Q7 (see Fig 2). Conversely, the combined sum of all the vertices made up by all components with five or fewer co-authors takes up more than 50% of all the vertices at Q1, but this combined sum only contributes 21% of the vertices by Q7 (see Fig 2). A similar phenomenon can be seen for the share of publications attributable to the largest component. In Q1, the combined sum of all components with five or fewer co-authors publishes more than 50% of all articles; whereas the largest component only publishes 8.4% of the articles (see Fig 3). However, by Q3 the largest component takes a larger share of the publications than all the components with five or fewer co-authors combined; and starting with Q5 (2000–2004), the single largest component publishes more than 50% of all the articles (Fig 3).

## Discussion

Using traditional bibliometric indexes of productivity such as number of peer reviewed publications and number of published authors, our results showed a steady net increase in the productivity (number of publications) of scientific research from the first Q (1980–1984) to the most recent Q (2010–2014). Given that the period of time covered in our study overlaps but also extends three years beyond the most recent bibliometric study on EDs (i.e., [11]), our findings contradict Clinton [10] in that we can affirm that the increased interest in the scientific study of EDs that started in the 1960s remains strong beyond the first decade of the 21^st^ Century (see also [12, 21, 22, 28]).

The progressive increase in the number the scientific articles published during the overall 35-year period studied is likely the combined effect of a number of factors: (a) a possible increase in the global prevalence of EDs [29, 30], (b) increase in the awareness or recognition of eating disorders as a grave public health problem [31], and (c) other factors that may influence the augmentation of scientific activity regardless of scientific discipline [32]. Whereas our analyses cannot parse out all the specific factors that contributed to the noted increase in scientific productivity in ED, we can provide important observations about the extent to which individual and collaborative efforts may have accounted for the noted diachronic increase in the overall scientific activity in ED [12].

We can confidently rule out changes in individual author productivity (articles per author) as responsible for the noted augmentation in scientific activity. First, the Transience Index (percentage of authors with a single publication), as well as the proportions of authors with 2 to 9 and more than 10 publications did not substantively vary from Q2 to Q7 (see Table 5). In fact, the noted increase in overall scientific productivity occurred in lieu of a gradual decline in the ratio of articles to authors.

Over the 35-year period examined, there was a 10-fold increase in the number of published authors and 6-fold increase in the number of publications per Q. Thus, we can assert with confidence that the number of published authors correlated positively with the number of publications. This increase in the number of authors was the result of author incorporation rates (newcomers) that outpaced author attrition rates (terminators), and a small but sustained increase in the longevity of the existing authors (continuants).

Among the many possible factors that may have enabled or contributed to the noted remarkable growth in the net number of publications and authors per Q, we can point to the transformation or development of collaborations or author networks. To our knowledge, our study is the first to document within ED the dominant tendency in science toward multi-authored publications in detriment of sole authorships [33]. This can be observed in the increase in the percentage of authors who never published single authored articles, which grew from 79% to almost 96%, as well as in the decline of both authors with only single authored publications (14% to 2%) and authors with both single and co-authored publications (7% to 2%) (see Table 6). Thus, it is not surprising that the overall increase in the number of scientific publications observed occurred in parallel with the growth of works produced in collaboration. This noted tendency toward collective authorships, or increase in the average number of signatures per article, replicates results found in other disciplines including drug addiction research (e.g., [34]). For example, transience index values higher than 70% have been observed in Biomedicine and the Social Sciences [35–38].

The observed time-dependent growth in the number of signatures per article might be a response to the increasing need or demand to investigate phenomena from an interdisciplinary perspective to ensure a more complete and comprehensive advancement of scientific knowledge [39–41]. A high degree of collaboration within a discipline could be an indicator of scientific strength and vibrancy, as collaborations may improve both the quantity [35] and quality [42] of the research findings, elements that are all indispensable for the development of science and the advancement of knowledge [43]. Unfortunately, our database does not allow us to investigate with precision the extent to which the collaborations noted in ED are interdisciplinary, a question that deserves exploration in future research.

The network analyses combined with the collaboration measures provide a number of interesting observations. Most notably, the results show that the detected interest in collaborative work leads to the proliferation of research components with a high degree of clustering (i.e., high interconnectivity within individual components), in part because most article collaborations involve a reduced number of coauthors within relatively small components. In addition, our analyses suggest the presence of a mature, co-author network of ED that is similar other well developed and stablished networks across various disciplines.

Although the number of edges grew proportionally at a higher rate than the number of authors, the density of the components decreased over time. Nonetheless, the average distance among reachable pairs in the last 5 Qs is similar to those reported by Bordons and collaborators [44] in a study that examined social network activity between 2006–2008 within various disciplines. This means that the average distance, or minimum number of pathways need to connect two unlinked authors is relatively short within the ED network (see also [45]).

According to Fatt and collaborators [46] the component formed by the largest number of authors (or giant component) might be considered the network nucleus of any given of research area. The relative size of the largest component matters, as the size is an indicator of the existence of a “core” field in the research community, and an indicator that knowledge flows a relatively fast pace (see [13]). In general, there appears to be strong consensus that well- formed giant formed giant components include more than 40 to 90% of the vertices [13]. Within the present study we have noted and illustrated that not only the nucleus grew considerably from 6% (1980–1984) to over 50% (2010–2014), but that the producivity attributed to the nucleus surpassed the 50% mark by the fifth Q (1995–1999).

The high clustering coefficients (0.91 and 0.94) within each Q suggest that throughout the entire 35-year period there is high interconnectivity between the vertices, which is made possible by a high percentage of small clusters and the existence of structures that recreate the small-world phenomenon (The small-world phenomenon refers to the often found fact that any two individuals within any given social network are most likely connected by a short sequence of intermediate acquaintances) within the giant and larger components [26]. As reported by others [47–49], authors tend to create collaboration groups that connect fully all of its members. It has been argued that high clustering within the Health and Social Sciences is a necessary condition for the advancement of knowledge [50]. Our findings are similar to those reported by Barabásis and collaborators [15] with regard to the field of Neuroscience, or by Bordons and collaborators [44], for various disciplines.

### Limitations

Although our search approach yielded the largest sample or ED-related articles to date, our sample likely excluded a not insignificant number on non-English journals not covered by the WoS Core Collection database. This limitation may have been lessened by our decision to also mine Scopus, which includes a wide coverage of journals in languages other than English [51]. It is also possible but unlikely that we may have failed to include journals that might be covered by other databases such as PsycINFO. Nonetheless, WoS Core Collection and Scopus are the databases used most often in bibliometric studies because they include scientific literature of greater international impact [52] and other national databases play a more peripheral role in communicating the scientific literature [53].

Our search strategy may have been narrower than we portray it because we limited our search keywords to feeding and eating disorders terms, but excluded other eating disorder-related terms such as “restrictive eating” or “purging behaviors” or “body image disturbance”. We therefore checked the impact of leaving out the terms noted above and found we potentially missed adding up to 154 articles to our final sample (or a 0.35% net increase, assuming none of the 154 articles were redundant with any of the articles already in our sample). Our search strategy may have also been wider than it needed to be. That is, some researchers may find that our inclusion of ‘pica’ and ‘rumination’ disorders, which accounted for about 3% of all the articles in our search, may not be highly relevant to the ED field. However, these disorders are listed in the DSM-5 and previous DSM versions alongside anorexia and bulimia under the “feeding and eating disorder” categorization. Thus, while acknowledging our search could have been broader or more comprehensive in some respects, and narrower in others, we believe that the sum of potentially missed articles and perhaps extraneous articles is too small to have made much of a difference in the overall pattern of findings.

Another limitation is that we could not address or describe the likely interdisciplinary makeup of the ED co-author network, and thus cannot speak to the extent to which attempts to advance knowledge in ED contributed to the observed increase in collaborative work over time.

### Future research

As possible future lines of research, we believe that it would be important to conduct more in-depth analyses of the evolution of specific areas within ED contrasted against each other, as well as comparing the relative penetration of ED research across related sciences such as Health Sciences, Education Sciences or Social Sciences, or across associated disciplines and bodies of knowledge, such as epidemiology, clinical, pathology and prevention. It might also be important to examine in greater depth the factors or motivations that drive researchers to collaborate, as for example the hypothesized desire and need to understand phenomena fully by incorporating interdisciplinary perspectives.

We hope our investigation inspires other lines of inquire, such a more direct examination of how collaborative research across investigators and research centers might be associated with external funding success, or with the ability to publish in high impact journals. Similarly, of great interest to the profession, at a time when interdisciplinary collaborations have become a buzz word, it would important to study how social networks contribute or become barriers to promotion or tenure within universities and research institutions.

Finally, EDs, once conceptualized as “culture-bound” syndromes rooted in Western culture [54,55], are or have become more prevalent among non-Western cultural groups and nations that previously thought [56]. Although not within the scope of the current study, it would be interesting and insightful to investigate the extent to which the realization that EDs are not phenomena circumscribed to Anglo/Western cultures is the result of increased collaborations across investigators across borders, or vice versa [30].
